# A Chiral Metal—Organic Framework Prepared on Large-Scale for Sensitive and Enantioselective Fluorescence Recognition

**DOI:** 10.3390/molecules28124593

**Published:** 2023-06-07

**Authors:** Xin-Mei Zhang, Yan-Mei Bai, Lu-Lu Ai, Fang-Hui Wu, Wei-Long Shan, Yan-Shang Kang, Li Luo, Kai Chen, Fan Xu

**Affiliations:** 1 School of Chemistry and Chemical Engineering, Anhui University of Technology, Maanshan 243002, China; zxm13295603905@163.com (X.-M.Z.); bym1101074898@163.com (Y.-M.B.); a8156162496158@163.com (L.-L.A.); wfhwfh@ahut.edu.cn (F.-H.W.); wlshan@ahut.edu.cn (W.-L.S.); 2 School of Chemistry and Life Sciences, Suzhou University of Science and Technology, Suzhou 215009, China; 3 Collaborative Innovation Center of Atmospheric Environment and Equipment Technology, Jiangsu Key Laboratory of Atmospheric Environment Monitoring and Pollution Control, Nanjing University of Information Science & Technology, Nanjing 210044, China; catqchen@163.com; 4 SJTU SMSE—Mingguang Joint Research Center for Advanced Palygoskite Materials, Mingguang Mingyao Attapulgite Industry Technology Co., Ltd., Chuzhou 239400, China; xufan921222@163.com

**Keywords:** chiral metal-organic framework, luminescence, enantioselectivity, amino acid, chiral recognition

## Abstract

MOF-based luminescent sensors have garnered considerable attention due to their potential in recognition and discrimination with high sensitivity, selectivity, and fast response in the last decades. Herein, this work describes the bulk preparation of a novel luminescent homochiral MOF, namely, [Cd(*s*-**L**)](NO_3_)_2_ (MOF-**1**), from an enantiopure pyridyl-functionalized ligand with rigid binaphthol skeleton under mild synthetic condition. Except for the features of porosity and crystallinity, the MOF-**1** has also been characterized with water-stability, luminescence, and homochirality. Most important, the MOF-**1** exhibits highly sensitive molecular recognition toward the4-nitrobenzoic acid (NBC) and moderate enantioselective detection of proline, arginine, and 1-phenylethanol.

## 1. Introduction

Due to rising concern about environmental issues and human health, chemical recognition for industrial and pharmaceutical pollutants has become a hot-topic research focus over the last decades [1,2]. Particularly in domestic water, both organic pollutants and heavy-metal anions will cause irreversible organ damage when accumulated to a certain threshold in the food chain [3,4]. Therefore, the development of a simple and fast-responses method to recognize these pollutants is a vital but challenging task. Despite of the attentive study and great successes in discrete complexes as chemical sensors, their practical applications have been hindered by the limited recyclability and synthetic challenges [5,6,7,8,9,10]. The crystalline and luminescent metal-organic frameworks (MOFs) have attracted huge interest in recent years because of their potential performance in simultaneous recognition and separation [11,12,13,14].

Asa promising alternative, these crystalline MOFs with periodic network structures are usually assembled from inorganic nodes and organic linkers, equipped with well-defined pore and channel structures. The superiority of luminescent MOFs for selective recognition to other report sensory materials is attributed to an additional mechanism for exclusion of unwanted species in selective sensing [15,16]. Mostly, the reason behind using luminescent MOFs for recognition and discrimination is closely related to preferential interactions between the guest species and the pore surfaces [16,17,18,19]. The structure-related properties of these MOFs, such as chemical stability, pore functionality, and structural tunability, maximize the promotion of dynamic response for various guest molecules/ions [20]. Thus, the key prerequisites to achieve high selectivity and sensitivity are the acquisition of desirable architectures in construction of MOFs [21].

Notably, rational functionalization of organic ligands as linkers is deemed to be an essential factor for design of corresponding functional materials [22,23]. Occasionally, the luminescent intensity of MOFs originating from organic linkers is unstable during the transmission and amplification of recognition signals [24]. To improve luminescence properties, diverse coordinated linkers with conjugated aromatic units are commonly used for their intrinsic optical and electronic performance [25,26,27,28,29]. By comparison, N-containing ligands, as exemplified by pyridine and their derivatives, stand out for their defined coordination fashion and strong coordination abilities [30,31]. Moreover, the preference of N-donating ligands to bond with transition-metal ions in a highly organized manner assures the large-scale synthesis and precise control of MOFs with special structure [32,33,34]. More than that, if chirality is integrated into crystalline architecture using enantiopure N-containing ligands, target MOFs will exhibit preferential interaction with one of the enantiomers rather than another one [35,36]. The inherent features of such strategies in constructing homochiral MOFs open up unique and valuable possibilities to multifunctional materials with diversified specific properties.

## 2. Results and Discussion

### 2.1. Synthesis and Characterization

In this context, we aim to present a fluorescent MOF-based sensor comprised of chiral N-containing ligands derived from pyridyl-functionalized binaphthol (BINOL). This distinctive framework is fluorescent and homochiral, which is expected to have good enantioselective performance in chemical recognition [37]. Colorless octahedral crystals of composition [Cd(*s*-**L**)](NO_3_)_2_ (MOF-**1**) {*s*-L = (s)-4,4′,4″,4‴-(2,2′-diethoxy-[1,1′-binaphthalene]-4,4′,6,6′-tetrayl)tetrapyridine} (CCDC2254999 (**1**)) were formed on a large scale by coordination-driven self-assembly of Cd(NO_3_)_2_·4H_2_O and *s*-**L** in 1,4-dioxane/H_2_O at 30–40 °C for several hours-. The phase purity of bulk samples of MOF-**1** was confirmed by comparison of its observed and simulated powder XRD (PXRD) patterns. In addition, MOF-**1** was characterized by single crystal X-ray diffraction, infrared (IR) spectroscopy, thermal gravimetric analysis (TGA), and circular dichroism (CD) spectroscopy.

**Figure 1:**
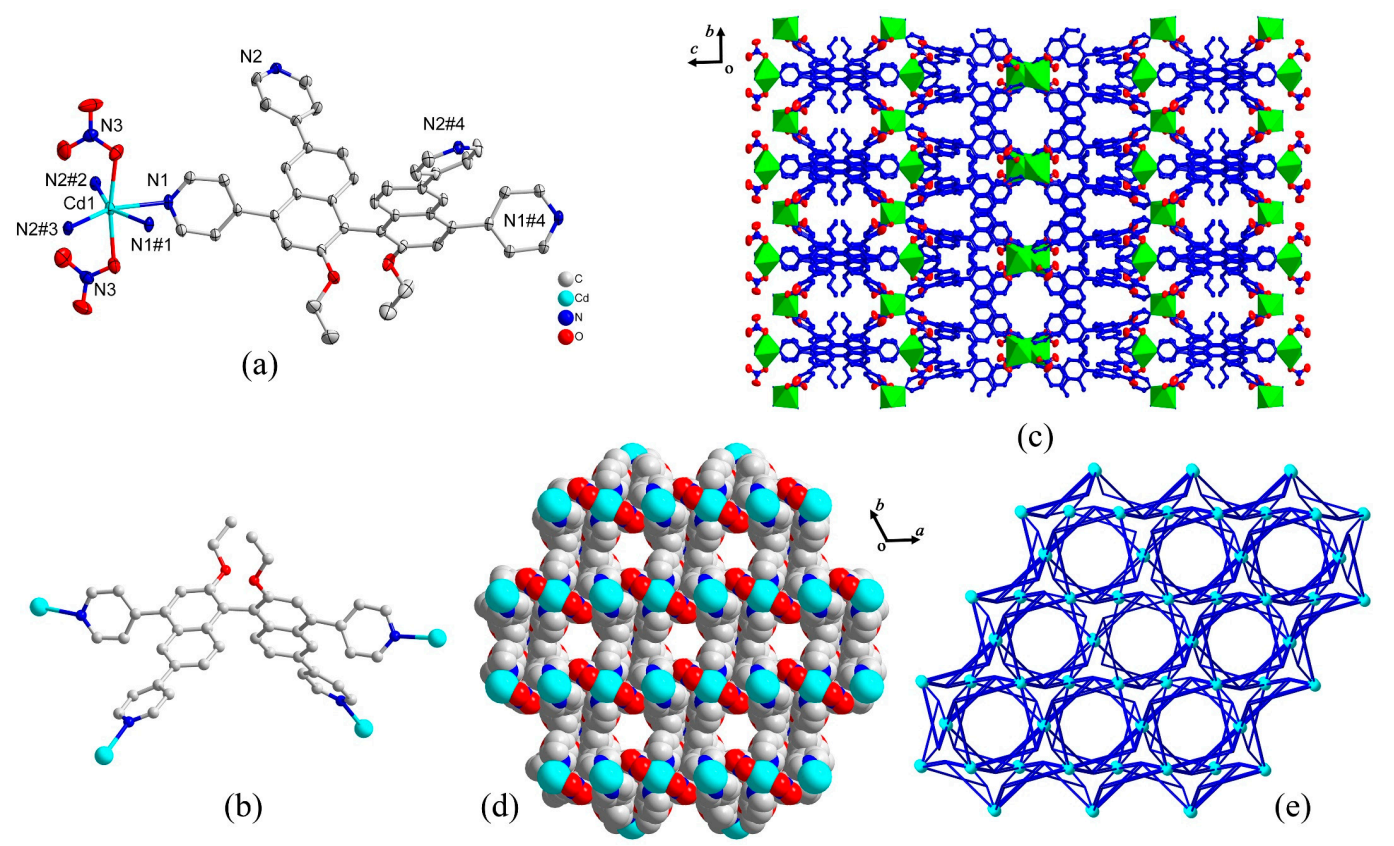
(**a**) Coordination environment of Cd^2+^ ion in MOF-**1**. (Symmetry codes: #1: 1-*x*, 1-*y*, *z*; #2: 2-*x*, 1-*x*+*y*, 1/3-*z*; #3: -1+*x*, *x*-*y*, 1/3-*z*; #4: 1-*x+y*, *y*, 1-*z*); (**b**) Coordination style of *s*-L ligand; (**c**) Polyhedral view of 3D framework of MOF-**1** from *a* axis; (**d**) Space-filling of the porous 3D framework of MOF-**1** with 1D hexagon channel; (**e**) (4,4)-connected topology. Hydrogen atoms and solvent molecules are omitted for clarity.

Single crystal X-ray diffraction analysis reveals that MOF-**1** crystallizes in the hexagonal *P*_64_ space group with a Flack parameter of −0.036(12), indicating the enantiomeric purity of the single crystal. The asymmetric unit of MOF-**1** contains one Cd^2+^ ion, half of **L** ligand and one nitrate ion, with the guest solvent molecules squeezed. The central Cd^2+^ ion is hexa-coordinated by four pyridyl nitrogen atoms from four **L** ligands and two oxygen atoms from two NO_3_^−^, displaying slightly distorted [CdN_4_O_2_] octahedral coordination geometry (Figure 1a). The *s*-**L** ligand adopts a tetradentate coordination mode to connect four metal centers via pyridine units (Figure 1b), thus forming a novel three-dimensional (3D)MOF long the *a* axis. The *s*-**L** ligand in MOF-**1** is twisted with an axially chiral *S*-conformation because of the steric hindrance between the naphthyl and ethoxy groups, and the corresponding dihedral angle within the fixed structure is ca. 97.45. As shown in Figure 1d, the MOF-**1** possessed 1Dregular hexagonal channels about 9.0 Å in diameter along the *c* axis (Figure 1d). The total solvent-accessible volume of MOF-**1** is estimated to be 27.3%, calculated by using PLATON. To better understand the intrinsic structure, MOF-**1** was analyzed by TOPOS 4.0 program 30. Each Cd^2+^ ionandeachligand simplify to a 4-connected node, respectively, and thus the whole framework could be regarded as binodal (4,4)-connected topology with a point symbol of (4^3^·8^2^).

**Figure 2:**
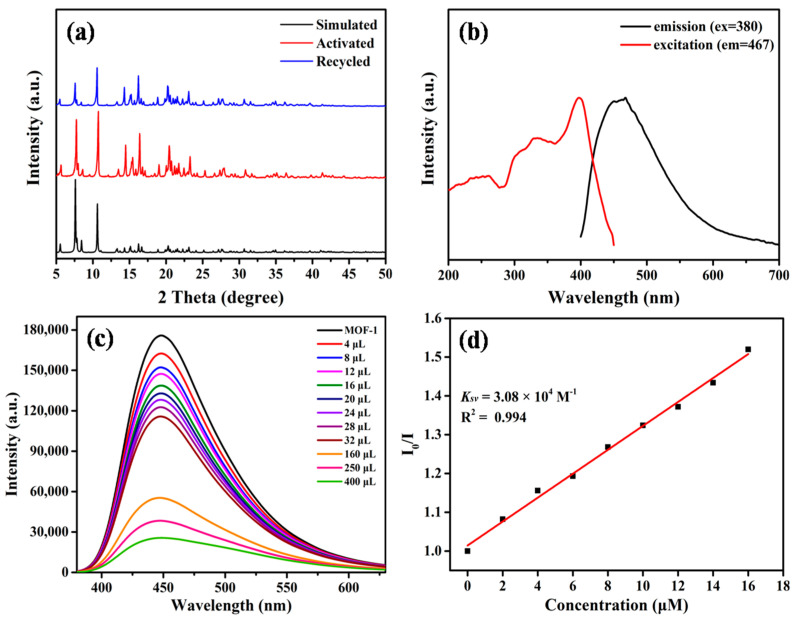
(**a**) The PXRD patterns of MOF-**1**; (**b**) Normalized solid-state emission and excitation spectraof MOF-**1**; (**c**) Luminescent emission spectra of MOF-1 in aqueous solution with different concentrations of NBC at room temperature (λ_ex_ = 380 nm); (**d**) Relationship between the quenching effect (*I*_0_/*I*) and the molar concentration of NBC (Q) (experimental conditions: aqueous solution, λ_ex_ = 380 nm, λ_em_ = 446 nm, and room temperature).

The Fourier transform-infrared (FT-IR) spectrum of MOF-1 showed strong peaks at 1604 cm^−1^ and 1382 cm^−1^, which can be attributed to the characteristic peaks of naphthalene and pyridine rings. Furthermore, the stretching vibration adsorption of typical C-O in aromatic ether appears as a middle peak at 1223 cm^−1^ (Figure S6). Framework stability of MOF-**1** was estimated before practical application in complicated actual conditions. The water stability of MOF-**1** was validated by PXRD measurements (Figure 2a). After immersion in water for two weeks, all the observed diffraction peaks are nearly identical to those of MOF-**1** untreated, demonstrating the high stability of MOF-**1** under neutral aqueous solution. The TGA curve of MOF-**1** revealed the removal of solvent molecules at 80–150 °C, and the decomposition of crystal framework at 300 °C (Figure S1). According to the analysis of single-crystal structure, there are no solvents or water molecules that coordinate to Cd ions. Thus, the weight loss in the first stage is a consequence of the liberation of free solvent molecules. The permanent porosity of activated MOF-**1** was examined by N_2_ adsorption measurements at 77 K, and the BET surface areas were found to be 290.2 m^2^/g (Figures S2 and S3).

The solid-state luminescence spectra of MOF-**1** shows only one strong emission peak at 467 nm upon excitation at 380 nm, ascribed to intra ligand electron transitions (Figure 2b). The strong emission indicates that MOF-**1** is atypical bluish-light material calculated with CIE coordinates. Considering its relatively chemical stability and good fluorescence performance, the luminescence sensing activities of MOF-**1** in aqueous solutions were investigated. The crystals of MOF-**1** were carefully ground to a powder and well dispersed in aqueous solution with the assistance of ultrasound. Fluorescence emission of the given suspension appeared at a similar position. As expected, 4-nitrobenzoic acid (NBC)as a toxic and explosive molecule, common in waste water, has a negative impact on the fluorescence intensity of the suspension of MOF-**1**. To further quantitative analyze the luminescence response of the MOF-**1** to NBC, a luminescence titration experiment was carried out by gradually increasing the concentration of NBC in the suspension. As depicted in Figure 2c, it was obvious that a certain linear relationship exists between the quenching effect and the amount of NBC in the low concentration. The quenching efficiency was evaluated from the traditional Stern-Völmer equation: *I*_0_/*I* = *K_sv_*[*Q*]+1, where *I*_0_ is the luminescence intensity of the initial suspension of MOF-**1**, and *I* is the intensity of the suspension resulting from the addition of the analyte; [*Q*] is the molar concentration of NBC. The slope of the equation (*K_sv_*) represents the quenching constant. The *K_sv_* value and the limited of detection (LOD) were calculated to be 3.08 × 10^4^ M^−1^ and 24 ppm (Figure 2d), respectively, which reveal that MOF-**1** has excellent sensitivity to recognize NBC in aqueous solution [38,39,40,41,42,43].

### 2.2. Enantioselective Fluorescent Sensing

**Figure 3:**
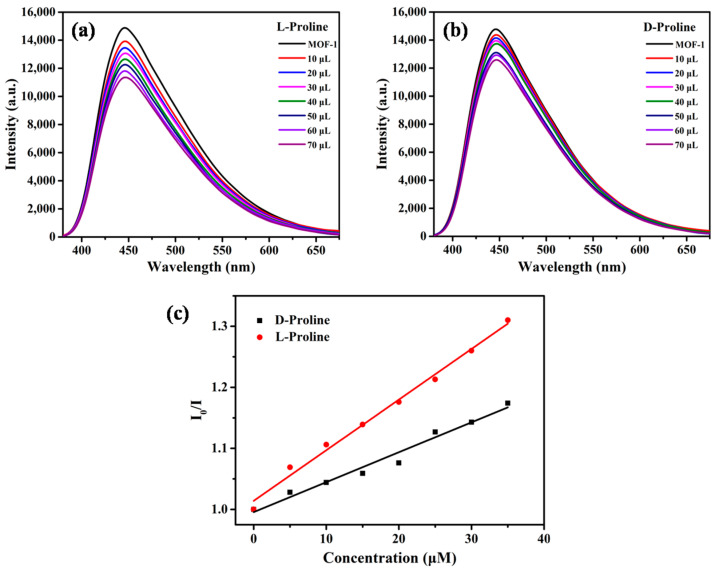
Fluorescence emission spectra of MOF-**1** (1.0 × 10^−5^ M in water) upon titration with: (**a**) *L*-Pro (1.0 × 10^−2^ M); and (**b**) *D*-Pro (1.0 × 10^−2^ M); (**c**) the Stern-Völmer plot.

**Figure 4:**
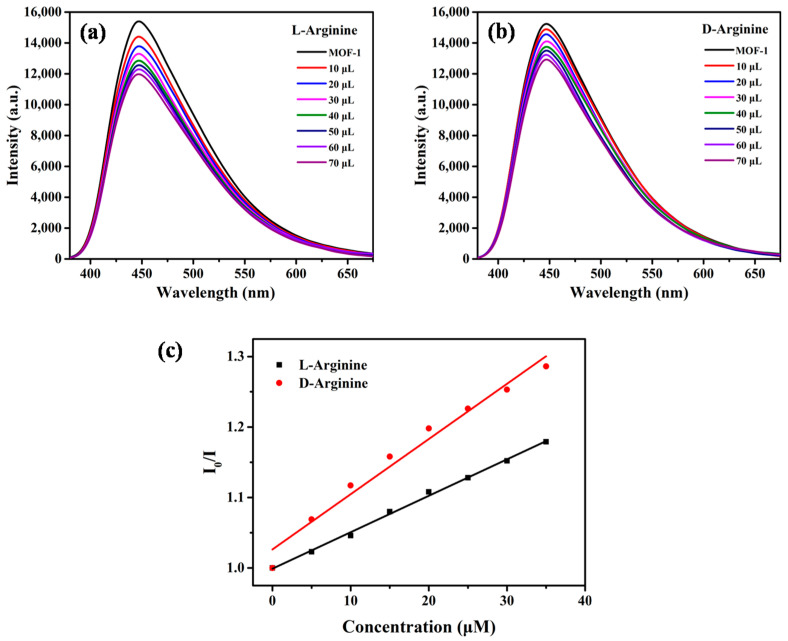
Fluorescence emission spectra of MOF-**1** (1.0 × 10^−5^ M in water) upon titration with: (**a**) *L*-Arg (1.0 × 10^−2^ M); and (**b**) *D*-Arg(1.0 × 10^−2^ M); (**c**) the Stern-Völmerplot.

**Figure 5:**
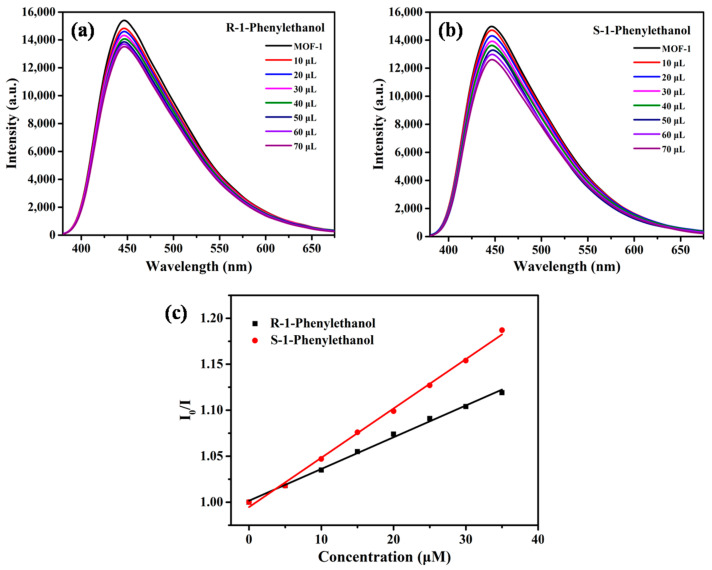
Fluorescence emission spectra of MOF-1 (1.0 × 10^−5^ M in water) upon titration with: (**a**) *R*-1-Phenylethanol (1.0 × 10^−2^ M); and (**b**) *S*-1-Phenylethanol (1.0 × 10^−2^ M); (**c**) the Stern-Völmerplot.

To confirm the homochirality of MOF-**1**, the CD spectrum of bulk crystal was measured in the solid state (Figure S5). The CD signals of the crystalline state were accordant with those of the corresponding enantiomer of ligand, indicating it senantiopurity and chirality derived from the chial 1,1′-naphthyl skeletons. The characteristics of the chiral channels, functional ethoxy group, and good fluorescence performance prompted us to further explore the enantioselectivity of MOF-**1** in aqueous solution. When MOF-**1** was treated with *D*/*L*-Proline (Pro), the emission position of the suspension stayed at 446 nm with no obvious translocation. The fluorescence intensity was decreased along with the *D*- and *L*-enantiomers affiliation, but the decrease rate caused by *L*-Pro was much greater than that by *D*-Pro, clearly manifesting enantioselectivity in the fluorescence recognition (Figure 3a,b). In addition, the fluorescence quenching of *D* and *L* enantiomers at 446 nm also follows the linear relationship of the Stern-Völmer equation. Figure 3c shows Stern-Völmer plots for MOF-1 (1.0 × 10^−5^ M) in the presence of *L*- and *D*-Pro in aqueous solution. The association constants *K_sv_* were calculated to be 718.59 M^−1^ with *L*-Pro and 458.54 M^−1^ with *D*-Pro, giving an enantioselectivity factor *K_sv_*_(MOF-1−L)_/*K_sv_*_(MOF-1−D)_ of 1.69. These results encouraged us to further study the enantioselectivity of MOF-**1** toward amino acids. Interestingly, the microcrystal MOF-**1** dispersed in the suspension, displayed enantioselective binding with *L*-Arginine (Figure 4). The selectivity factor *K_sv_*_(MOF-1−L)_/*K_sv_*_(MOF-1−D)_ was determined as 1.51. The enantioselectivity probably decreased because the guanidyl group of Arginine interferes with host–guest interaction in the chiral cavity of MOF-**1**. More importantly, MOF-**1** can also enantioselectively recognize1-Phenylethanol, a widely used edible flavoring. The relative intensity of *I*/*I*_0_ at 446 nm can also be linearly fitted with the concentration of the analyte (Figure 5). Based on the Stern-Völmer equation, MOF-**1** exhibited comparable enantioselectivity toward the 1-Phenylethanol quencher with a *K_sv_*_(MOF-1−S)_/*K_sv_*_(MOF-1−R)_ ratio of 1.55.The sensitivity of MOF-**1** essentially benefited from the preconcentration of the channels, which allows these small molecules to easily access the binding sites in low concentration. Furthermore, the fluorescence quenching behaviors were probably traceable to the supramolecular interactions between analyte with MOF-**1** in the ground. It should also be pointed out that the crystallinity of MOF-**1** remains intact after the chiral recognition process confirmed by PXRD patterns. The above results suggest that MOF-**1** as a new fluorescence sensor has the potential to perform enantioselective detection and discrimination in water.

## 3. Materials and Methods

### 3.1. General

All of the chemicals and solvents were purchased from commercial sources and used as supplied unless otherwise mentioned. The ligand of (s)-4,4′,4″,4‴-(2,2′-diethoxy-[1,1′-binaphthalene]-4,4′,6,6′-tetrayl)tetrapyridine(*s*-L) was synthesized as modified in the literature [44]. Powder X-ray diffraction (PXRD) data were recorded on a Bruker D8 discover powder diffractometer with Cu Kα radiation. The calculated PXRD pattern was produced using the SHELXTL-XPOW program and single crystal reflection data. Thermogravimetric analysis (TGA) measurements were conducted under nitrogen atmosphere at a heating rate 10 °C/min with a Shimadzu DTG-60H. Elemental analyses were performed on an Elementar Vario EL III analyzer. IR spectra of the solid samples (KBr tablets) in the range 400–4000 cm^−1^ were recorded on a Nicolet Magna 750 FT-IR spectrometer. The N_2_ adsorption/desorption isotherms were measured volumetrically using a Micromeritics ASAP 2020 surface area.UV/vis absorption spectra were recorded on a TU-1810 UV/vis spectrophotometer. The solid cicular dichroism (CD) spectra was obtained by using a BRIGHTTME Chirascan CD spectrometer. Single-crystal XRD data of MOF-**1** were all collected on a Bruker APEX area-detector X-ray diffractometer with Mo-Kα radiation (λ = 0.71073 Å). The empirical absorption correction was applied by using the SADABS program. The structures were solved using direct methods, and refined on *F*^2^ by a full-matrix least-squares method. All calculations were carried out with the SHELXTL program. Disordered solvent molecules that could not be restrained properly were removed using the SQUEEZE routine in all data sets. Crystal data, data collection parameters, and the results of the X-ray diffraction studies are given in Table S1.

### 3.2. Synthesis of [Cd(s-L)](NO_3_)_2_(MOF-1)

A mixture of Cd(NO_3_)_2_·4H_2_O (15.4 mg, 0.28 mmol), *s*-L (46.0 mg, 0.07 mmol), 1,4-Dioxane (10.0 mL) and H_2_O (0.5 mL) in a capped vial (20 mL) was stand at 30–40 °C for several hours. Elongated octahedron crystals of MOF-**1** suitable for single-crystal X-ray diffraction were harvested after simple filtration, washed with water and activated with acetone. *Data for* MOF-**1**: yield: 56.7 mg, 90%. Elemental analysis calcd (%) for CdC_44_H_34_O_8_N_6_ (M = 887.17): C, 59.52; H, 3.83; N, 9.47. Found: C, 59.76; H, 3.85, N, 9.49. IR (KBr pellet, cm^−1^): *v* = 3446.9 (m), 2978.9 (w), 2427.4 (w), 1604.9 (s), 1587.0 (s), 1543.3 (m), 1488.0 (m), 1454.0 (w), 1382.3 (vs), 1342.1 (w), 1318.2 (w), 1223.8 (m), 1113.7 (w), 1065.9 (w), 1039.6 (w), 837.9 (w), 824.6 (m), 814.3 (m).

## 4. Conclusions

In summary, we have successfully prepared a novel luminescent MOF with good water stability through the coordination-directed assembly of homochiralpyridyl-functionalized precursor based on rigid binaphthol skeleton. The high-quality crystals can be achieved on a large-scale in a few hours, benefiting from its mild synthetic condition. The fluorescence titration experiments imply that MOF-**1** has good sensitivity to detect the organic pollutant NBC in water. Interestingly, the MOF-**1** was developed into a fluorescent probe for the chiral sensing of Proline and Arginine with moderate enantioselectivity. In addition, further investigation found that the MOF-1exhibits enantioselective molecule recognition toward 1-Phenylethanol. This work opens an avenue toward the design and synthesis of homochiral MOFs as a potential probe for luminescent sensing of pollutants and enantioselective detection of racemates in water.

## Data Availability

The data presented in this study are available in Supplementary Materials.

## Supplementary Materials

The following supporting information can be downloaded at: https://www.mdpi.com/article/10.3390/molecules28124593/s1, Figure S1: The TGA Curve of MOF-1; Figure S2: N2 adsorption isotherm of MOF-1; Figure S3: The BET plots of MOF-1; Figure S4: UV/Vis diffuse reflectance spectrum of MOF-1; Figure S5: CD spectra of s-L and MOF-1 in the solid at room temperature; Figure S6: The FT-IR spectrum of MOF-1; Figure S7: Fluorescence excitation spectra of MOF-1 (1.0 × 10^−5^ M in water) and in the presence of L-Pro (1.0 × 10^−2^ M) and D-Pro (1.0 × 10^−2^ M); Figure S8: Fluorescence excitation spectra of MOF-1 (1.0 × 10^−5^ M in water) and in the presence of L-Arg (1.0 × 10^−2^ M) and D-Arg (1.0 × 10^−2^ M); Figure S9: Fluorescence excitation spectra of MOF-1 (1.0 × 10^−5^ M in water) and in the presence of R-1-Phenyethanol (1.0 × 10^−2^ M) and S-1-Phenyethanol (1.0 × 10^−2^ M); Table S1: Selected materials for detection of LODs and Ksv of 4-nitrobenzoic acid; Table S2: Crystal data and structure refinement for MOF-1.

## References

[1] Gao R., Kodaimati M.S., Yan D. (2021). Recent advances in persistent luminescence based on molecular hybrid materials. Chem. Soc. Rev..

[2] You L., Zha D., Anslyn E.V. (2015). Recent advances in supramolecular analytical chemistry using optical sensing. Chem. Rev..

[3] Umapathi R., Sonwal S., Lee M.J., Mohana Rani G., Lee E.S., Jeon T.J., Kang S.M., Oh M.H., Huh Y.S. (2021). Colorimetric based on-site sensing strategies for the rapid detection of pesticides in agricultural foods: New horizons, perspectives, and challenges. Coord. Chem. Rev..

[4] Xu N., Zhang Q., Hou B., Cheng Q., Zhang G. (2018). A novel magnesium metal-organic framework as a multiresponsive luminescent sensor for Fe(III) ions, pesticides, and antibiotics with high selectivity and sensitivity. Inorg. Chem..

[5] Yu F., Chen Y., Jiang H., Wang X. (2020). Recent advances of BINOL-based sensors for enantioselective fluorescence recognition. Analyst.

[6] Crassous J. (2009). Chiral transfer in coordination complexes: Towards molecular materials. Chem. Soc. Rev..

[7] Thoonen S., Hua C. (2021). Chiral detection with coordination polymers. Chem.-Asian J..

[8] Lee M., Ok K.M. (2022). Chiral coordination compounds with exceptional enantioselectivity. J. Mater. Chem. C.

[9] Alizada M., Gul A., Oguz M., Kursunlu A.N., Yilmaz M. (2021). Ion sensing of sister sensors based-on calix[4]arene in aqueous medium and their bioimaging applications. Dyes. Pigment..

[10] Kursunlu A.N., Sahin E., Güler E. (2016). Cu(II) chemo sensor based on a fluorogenic bodipy-salophen combination: Sensitivity and selectivtity studies. J. Fluoresc..

[11] Dong J., Zhao D., Lu Y., Sun W.Y. (2019). Photoluminescent metal-organic frameworks and their application for sensing biomolecules. J. Mater. Chem. A.

[12] Raja Lakshmi P., Nanjan P., Kannan S., Shanmugaraju S. (2021). Recent advances in luminescent metal-organic frameworks (LMOFs) based fluorescent sensors for antibiotics. Coord. Chem. Rev..

[13] Zhao Y., Zeng H., Zhu X.W., Lu W., Li D. (2021). Metal-organic frameworks as photo luminescent biosensing platforms: Mechanisms and applications. Chem. Soc. Rev..

[14] Gomez G.E., Roncaroli F. (2020). Photofunctional metal-organic framework thin films for sensing, catalysis and device fabrication. Inorg. Chim. Acta.

[15] Lin X., Hong Y., Zhang C., Huang R., Wang C., Lin W. (2015). Pre-Concentration and energy transfer enable the efficient luminescence sensing of transition metal ions by metal-organic frameworks. Chem. Commun..

[16] Li B., Chen X., Hu P., Kirchon A., Zhao Y.-M., Pang J., Zhang T., Zhou H.-C. (2019). Facile fabrication of a multifunctional metal-organic framework-based sensor exhibiting exclusive solvochromic behaviors toward ketone molecules. ACS Appl. Mater. Interfaces.

[17] Yi F.Y., Chen D., Wu M.K., Han L., Jiang H.L. (2016). Chemical sensors based on metal-organic frameworks. Chempluschem.

[18] Lustig W.P., Mukherjee S., Rudd N.D., Desai A.V., Li J., Ghosh S.K. (2017). Metal-organic frameworks: Functional luminescent and photonic materials for sensing applications. Chem. Soc. Rev..

[19] Li H.Y., Zhao S.N., Zang S.Q., Li J. (2020). Functional metal-organic frameworks as effective sensors of gases and volatile compounds. Chem. Soc. Rev..

[20] Huang F.M., Wang M., Lin C., Wang J., Wu P. (2021). Luminescent metal-organic frameworks as chemical sensors based on “mechanism-response”: A review. Dalton Trans..

[21] Chen Z., Jiang H., Li M., O’Keeffe M., Eddaoudi M. (2020). Reticular chemistry 3.2: Typical minimal edge-transitive derived and related nets for the design and synthesis of metal-organic frameworks. Chem. Rev..

[22] Kalidindi S.B., Nayak S., Briggs M.E., Jansat S., Katsoulidis A.P., Miller G.J., Warren J.E., Antypov D., Cora F., Slater B. (2015). Chemical and structural stability of zirconium-based metal-organic frameworks with large three-dimensional pores by linker engineering. Angew. Chem. Int. Ed..

[23] Lu W., Wei Z., Gu Z.Y., Liu T.F., Park J., Park J., Tian J., Zhang M., Zhang Q., Gentle T. (2014). Tuning the structure and function of metal-organic frameworks via linker design. Chem. Soc. Rev..

[24] Xu N., Zhang Q., Zhang G. (2019). A carbazole-functionalized metal-organic framework for efficient detection of antibiotics, pesticides and nitroaromatic compounds. Dalton Trans..

[25] Mukhopadhyay A., Jindal S., Savitha G., Moorthy J.N. (2020). Temperature-dependent emission and turn-off fluorescence sensing of hazardous “Quat” herbicides in water by a Zn-MOF based on a semi-rigid dibenzochrysenetetra acetic acid linker. Inorg. Chem..

[26] Wang X., Lei M., Zhang T., Zhang Q., Zhang R., Yang M. (2021). A water-stable multi-responsive luminescent Zn-MOF sensor for detecting TNP, NZF and Cr_2_O_7_^2−^ in aqueous media. Dalton Trans..

[27] Zhao X., Wang S., Zhang L., Liu S., Yuan G. (2019). 8-Hydroxyquinolinate-based metal-organic frameworks: Synthesis, tunable luminescent properties, and highly sensitive detection of small molecules and metal ions. Inorg. Chem..

[28] Zhang Q., Lei M., Kong F., Yang Y. (2018). A water-stable homochiral luminescent MOF constructed from an achiral acylamide-containing dicarboxylate ligand for enantioselective sensing of penicillamine. Chem. Commun..

[29] Chandrasekhar P., Mukhopadhyay A., Savitha G., Moorthy J.N. (2016). Remarkably selective and enantio differentiating sensing of histidine by a fluorescent homochiral Zn-MOF based on pyrene-tetralactic acid. Chem. Sci..

[30] Jiao J., Dong J., Li Y., Cui Y. (2021). Fine-tuning of chiral microenvironments within triple-stranded helicates for enhanced enantioselectivity. Angew. Chem. Int. Ed..

[31] Thoonen S., Tay H.M., Hua C. (2022). A chiral binaphthyl-based coordination polymer as an enantioselective fluorescence sensor. Chem.Commun..

[32] Wang D., Zhang D., Han S.D., Pan J., Xue Z.Z., Li J.H., Wang G.M. (2019). A pillared-layer strategy to construct water-stable Zn-organic frameworks for iodine capture and luminescence sensing of Fe^3+^. Dalton Trans..

[33] Dong J., Zhou Y., Zhang F., Cui Y. (2014). A highly fluorescent metallosalalen-based chiral cage for enantioselective recognition and sensing. Chem. Eur. J..

[34] Dong J., Tan C., Zhang K., Liu Y., Low P.J., Jiang J., Cui Y. (2017). Chiral NH-controlled supra molecular metallacycles. J. Am. Chem. Soc..

[35] Gong W., Chen Z., Dong J., Liu Y., Cui Y. (2022). Chiral metal-organic frameworks. Chem. Rev..

[36] Xuan W., Zhang M., Liu Y., Chen Z., Cui Y. (2012). A chiral quadruple-stranded helicate cage for enantioselective recognition and separation. J. Am. Chem. Soc..

[37] Wanderley M.M., Wang C., Wu C.D., Lin W. (2012). A chiral porous metal-organic framework for highly sensitive and enantioselective fluorescence sensing of amino alcohols. J. Am. Chem. Soc..

[38] Karmakar M., Chattopadhyay S. (2020). Synthesis, structure and nitroaromatic sensing ability of a trinuclear zinc complex with a reduced Schiff base ligand: Assessment of the ability of the ligand to sense zinc ion. Polyhedron.

[39] Karmakar M., Basak T., Chattopadhyay S. (2019). penta-nuclear zinc(II) complex with a reduced Schiff base ligand: Assessment of its ability to sense nitroaromatics. New J. Chem..

[40] Dasgupta S., Zangrando E., Majumder I. (2017). A Comparative Study on “Turn-off” Fluorimetric Nitro Aromatic Detection Using a Class of Dinulear Zinc (II) Schiff Base Complexes. ChemistrySelect.

[41] Xin X.H., Lu W., Lu J., Xu J.G., Wang S.H., Zheng F.K., Guo G.G. (2018). A luminescent barium-based metal-organic framework: Synthesis, structure and efficient detection of 4-nitrobenzoic acid. Inorg. Chem. Commun..

[42] Mukherjee S., Ganguly S., Chakraborty A., Mandal A., Das D. (2019). Green Synthesis of Self Assembled Nanospherical Dysprosium MOFs: Selective and Efficient Detection of Picric Acid in Aqueous and Gas Phase. ACS Sustain. Chem. Eng..

[43] Qiao J., Liu X., Zhang L., Eubank J.F., Liu X., Liu Y. (2022). Unique Fluorescence Turn-On and Turn-Off−On Responses to Acids by a Carbazole-Based Metal−Organic Framework and Theoretical Studies. J. Am. Chem. Soc..

[44] Morohashi N., Iki N., Sugawara A., Miyano S. (2001). Selective oxidation of thiacalix[4]arenes to the sulfinyl and sulfonyl counterparts and their complexation abilities toward metal ions as studied by solvent extraction. Tetrahedron.

