# Supplementary material for: A Chiral Metal—Organic Framework Prepared on Large-Scale for Sensitive and Enantioselective Fluorescence Recognition
Source: Molecules. 2023 Jun 7;28(12):4593. doi: 10.3390/molecules28124593 (PMC10304791; doi:10.3390/molecules28124593)
Supplement: Supplementary file 1 [file molecules-28-04593-s001.zip › molecules-2366885-supplementary.pdf]

Supporting Information

A Chiral Metal−Organic Framework Prepared on Large-Scale for Sensitive and Enantioselective Fluorescence Recognition

Xin-Mei Zhang [1], Yan-Mei Bai [1], Lu-Lu Ai [1], Fang-Hui Wu [1], Wei-Long Shan [1], Yan-Shang Kang [1,*], Li Luo [2,*], Kai Chen [3] and Fan Xu [4]

[1] School of Chemistry and Chemical Engineering, Anhui University of Technology, Maanshan 243002, China
[2] School of Chemistry and Life Sciences, Suzhou University of Science and Technology, Suzhou 215009, China
[3] Collaborative Innovation Center of Atmospheric Environment and Equipment Technology, Jiangsu Key Laboratory of Atmospheric Environment Monitoring and Pollution Control, Nanjing University of Information Science & Technology, Nanjing 210044, China
[4] SJTU SMSE—Mingguang Joint Research Center for Advanced Palygoskite Materials, Mingguang Mingyao Attapulgite Industry Technology Co., Ltd., Chuzhou 239400, China
* Correspondence: kangys@ahut.edu.cn (Y.-S.K.); luolichem08@163.com (L.L.)

1. Characterizations

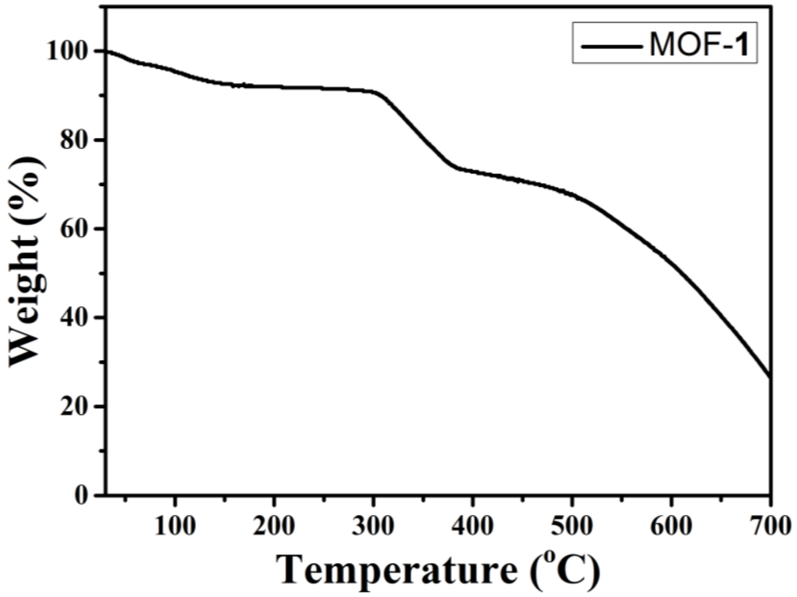

Figure S1 The TGA Curve of MOF-**1**.

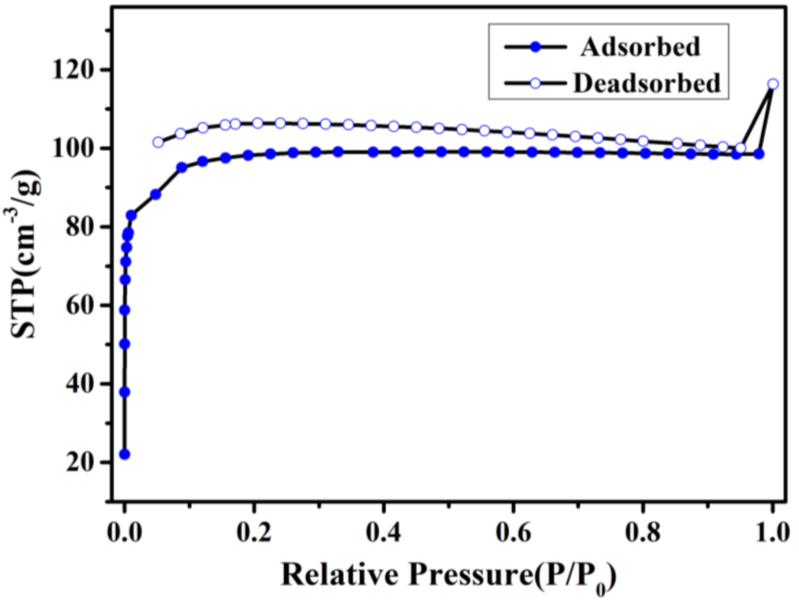

Figure S2 N_2 adsorption isotherm of MOF-**1**.

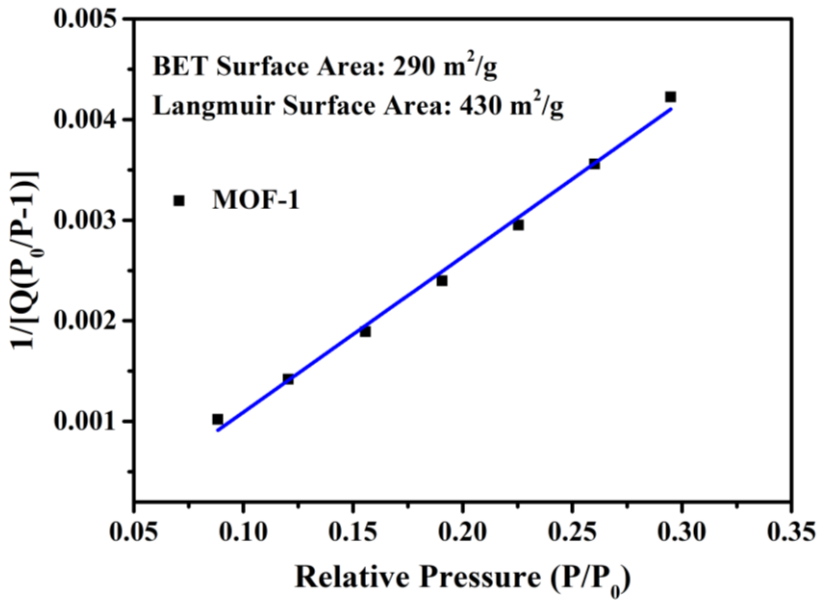

Figure S3 The BET plots of MOF-**1**.

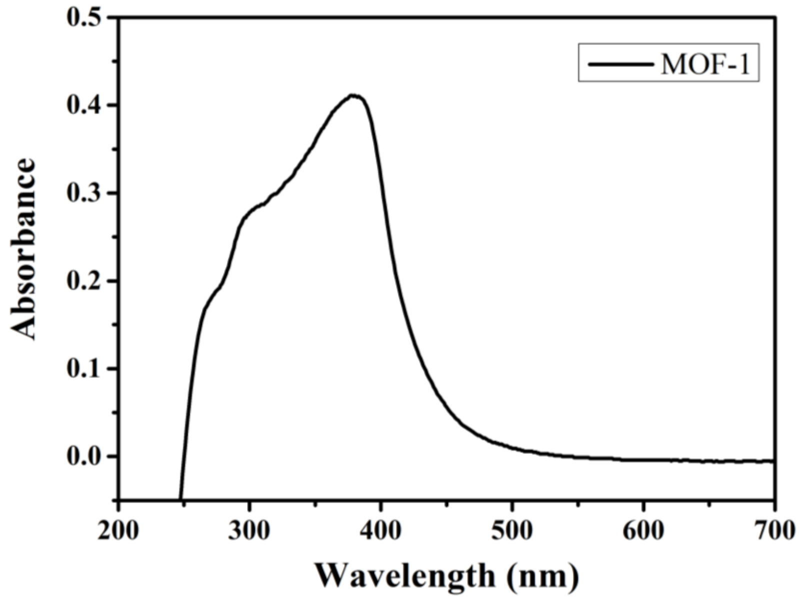

Figure S4 UV/Vis diffuse reflectance spectrum of MOF-**1**.

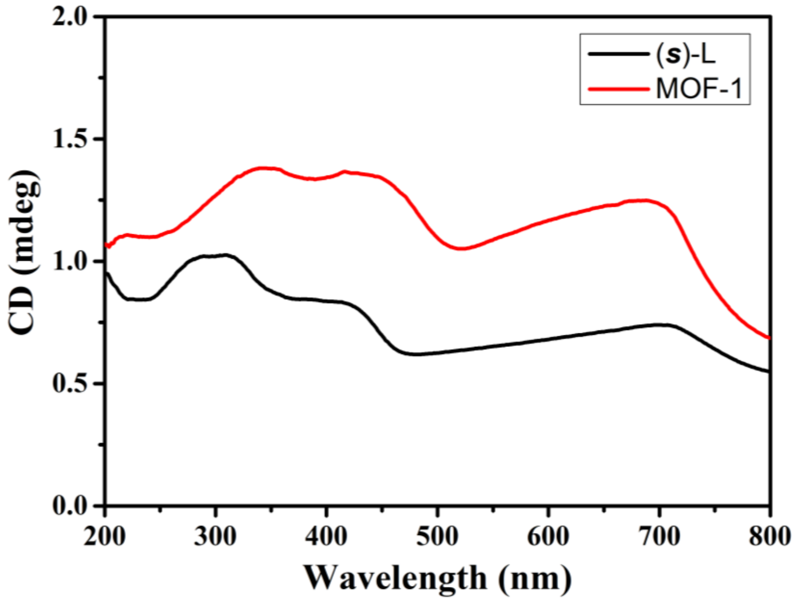

Figure S5 CD spectra of *s*-L and MOF-**1** in the solid at room temperature.

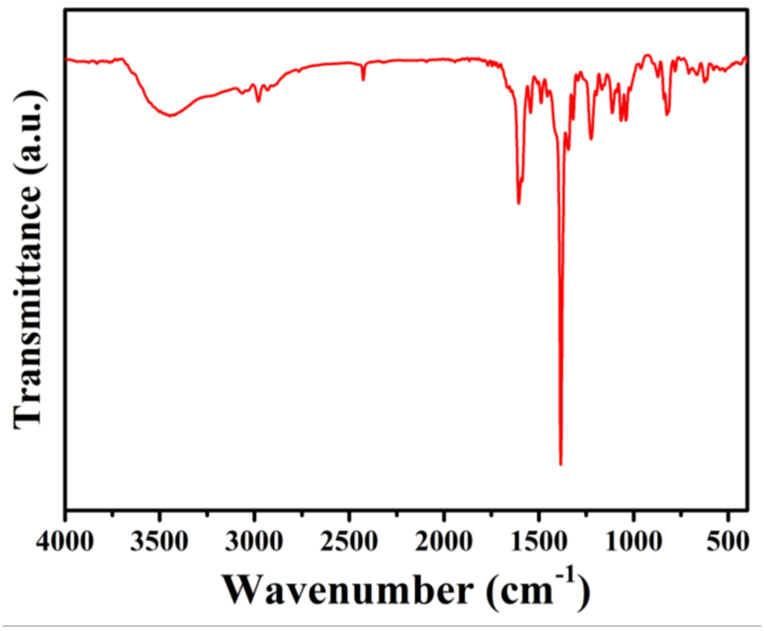

Figure S6 The FT-IR spectrum of MOF-**1**.

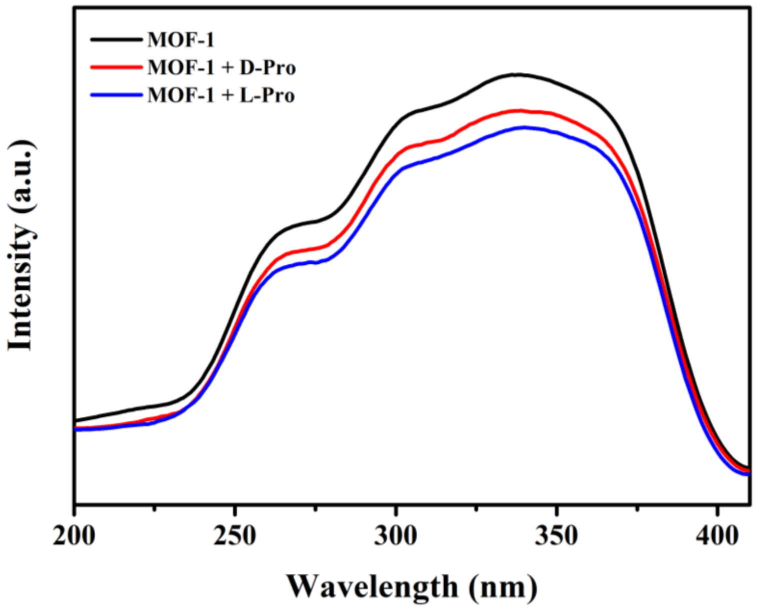

Figure S7 Fluorescence excitation spectra of MOF-**1** (1.0×10^{-5} M in water) and in the presence of *L*-Pro (1.0×10^{-2} M) and *D*-Pro (1.0×10^{-2} M).

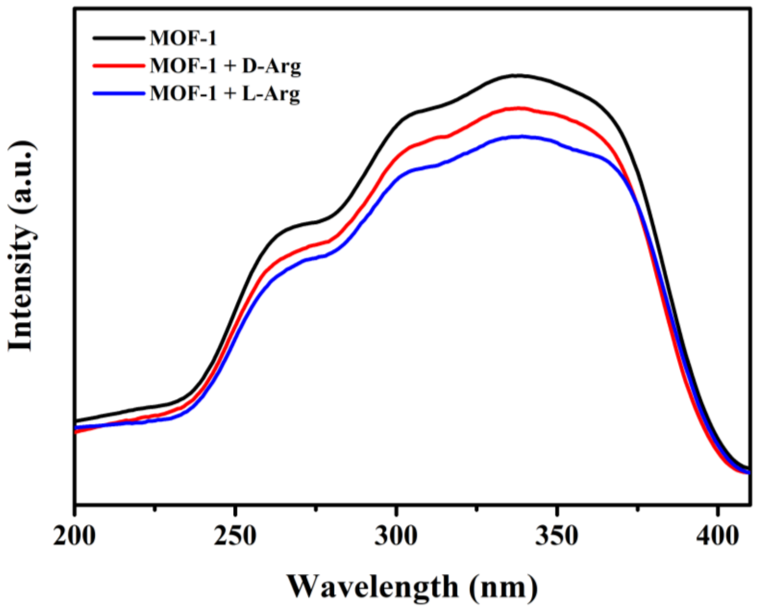

Figure S8 Fluorescence excitation spectra of MOF-**1** (1.0×10^{-5} M in water) and in the presence of *L*-Arg (1.0×10^{-2} M) and *D*-Arg (1.0×10^{-2} M).

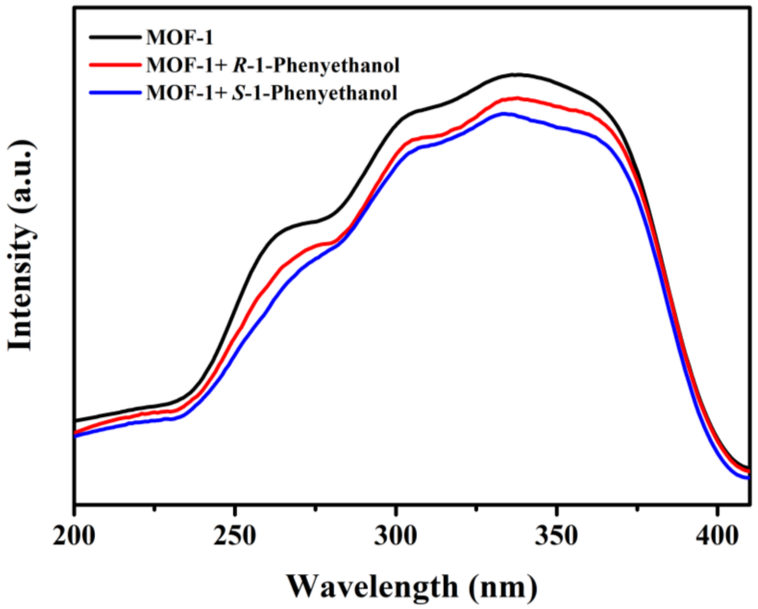

Figure S9 Fluorescence excitation spectra of MOF-**1** (1.0×10^{-5} M in water) and in the presence of *R*-1-Phenyethanol (1.0×10^{-2} M) and *S*-1-Phenyethanol (1.0×10^{-2} M).

Table S1. Selected materials for detection of LODs and Ksv of 4-nitrobenzoic acid

Material	LODs	Ksv (M^{-1})	Reference
$[Zn\{Zn(N_3)L\}_2]$ Complex	0.619 ppm	7.77 × 104	[38]
$[(u_{1,1}\text{-}N_3)_2\{(N_3)Zn(L)Zn\}_2Zn(L)]$ Complex	38 ppm	6.497 × 104	[39]
$[Zn_2(L)(OAc)_2]_2[Zn(NCS)_4]$Complex	not given	1.55 × 107	[40]
$[Ba_2(adda)_2(H_2adda)(H_2O)_2]n$	not given	not given	[41]
$[\{Dy(2N_3\text{-}TPA)_2(H_2O)(CH_3OH)\}]_n$	not given	8.3 × 104	[42]
JLU-MOF111	not given	325	[43]
MOF-1	24 ppm	3.08 × 104	This work

2. X-ray crystallography details

Table S2. Crystal data and structure refinement for MOF-1.

Empirical formula	$C_{44}H_{34}N_6O_8Cd$	
Formula weight	887.17	
Temperature	296(2) K	
Wavelength	0.71073 Å	
Crystal system	Hexagonal	
Space group	$P6_422$	
Unit cell dimensions	a = 13.403(3) Å	α= 90°.
	b = 13.403(3) Å	β= 90°.
	c = 47.819(12) Å	γ = 120°.
Volume	7439(4) $Å^3$	
Z	6	
Density (calculated)	1.188 Mg/m^3	
Absorption coefficient	0.491 mm^{-1}	
F(000)	2712	
Crystal size	0.2*0.3*0.15 cm	
Theta range for data collection	2.170 to 27.520°.	
Index ranges	-17<=h<=14, -16<=k<=17, -62<=l<=58	
Reflections collected	46336	
Independent reflections	5727 [R_{int} = 0.0480]	
Completeness to theta = 25.242°	99.80%	
Absorption correction	None	
Refinement method	Full-matrix least-squares on F^2	
Data/restraints/parameters	5727 / 614 / 279	
Goodness-of-fit on F^2	0.895	
Final R indices [I>2sigma(I)]	R_1 = 0.0366, wR_2 = 0.1146	
R indices (all data)	R_1 = 0.0441, wR_2 = 0.1213	
Absolute structure parameter	-0.036(12)	
Extinction coefficient	n/a	
Largest diff. peak and hole	0.544 and -0.475 e.$Å^{-3}$	

$^aR_1 = \Sigma||F_o| - |F_c|| $ (based on reflections with $F_o^2 > 2\sigma F^2$); $wR_2 = \{\Sigma[\omega(F_o^2 - F_c^2)^2]/\Sigma[\omega(F_o^2)^2]\}^{1/2}$; $w = 1/[\sigma^2 F_0^2 + (0.095P)^2]$; $P = [\max(F_o^2, 0) + 2F_c^2]/3$ (also with $F_o^2 > 2\sigma F^2$).
